# A randomized controlled trial of teriparatide for accelerating bone union and improving clinical outcomes in patients with pertrochanteric fracture fixation

**DOI:** 10.1038/s41598-025-03720-2

**Published:** 2025-06-03

**Authors:** Chotetawan Tanavalee, Srihatach Ngarmukos, Chavarin Amarase, Saran Tantavisut, Nonn Jaruthien, Aree Tanavalee

**Affiliations:** 1 Biologics for Knee Osteoarthritis Research Unit, Faculty of Medicine, Chulalongkorn University, Bangkok, Thailand; 2 Department of Orthopaedics, Faculty of Medicine and King Chulalongkorn Memorial Hospital, Chulalongkorn University, Bangkok, Thailand; 3 Hip Fracture Research Unit, Faculty of Medicine, Chulalongkorn University, Bangkok, Thailand

**Keywords:** Teriparatide, Pertrochanteric fracture, Union, Fixation, Health care, Medical research

## Abstract

This prospective, double-blind, placebo-controlled, randomized trial evaluated the effects of teriparatide following pertrochanteric fracture fixation on bone healing and clinical outcomes. Among 50 participants having fractures and undergoing surgical fixation, 25 were randomly assigned to each group to receive daily teriparatide or placebo for 12 weeks and were followed until 24 weeks postoperatively. The primary outcome was the radiographic bone union. The secondary outcome was clinical results, including Harris hip scores (HHS) and performance-based tests evaluated at the postoperative 2nd, 4th, 6th, 12th, and 24th weeks, and spine and contralateral hip bone mass density (BMD), comparing admission and the 24th week. There were no statistically significant differences in baseline characteristics, including age, sex, fracture classification, affected side, HHS, BMD, and blood test results. The mean and standard deviation of radiographic bone union time of the teriparatide and the placebo groups were 7.44 ± 3.34 weeks and 10.56 ± 4.98 weeks, respectively, with significant differences (*p*, 0.0083). From the 6th week, both teriparatide and placebo groups had significantly improved HHS (*p*, 0.008 and 0.0205, respectively) and time up-and-go test (TUGT) (*p*, 0.0348 and 0.0237, respectively). In the 24th week, the five-time sit-to-stand test (5 × SST) of teriparatide and placebo groups was significantly improved (*p*, 0.0013 and 0.0412, respectively). However, there were no differences in HHS, TUGT and 5 × SST between groups at all follow-up time points. In the 24th week, the teriparatide group had less decrease in spine, femoral neck, and total hip BMD from baseline to the placebo group; however, these differences were insignificant. In conclusion, a 12-week teriparatide administration following intertrochanteric fracture fixation significantly shortened the fracture healing time. Although there were no differences in improved clinical outcomes, the teriparatide group had less decline in BMD at 24 weeks than the placebo group.

**Supplementary Information:**

The online version contains supplementary material available at 10.1038/s41598-025-03720-2.

## Introduction

Hip fracture is a global health problem with a cost burden, and the incidence increases yearly due to the increase in the world’s elderly population and human life expectancy^1,2^. Worldwide, the number of hip fractures was 1.3 million in 1990, which increased to 178 million in 2019, and the global prevalence became 5614.3 cases per 100,000 population^3,4^. In Thailand, a report in 2017 showed a high incidence of 8446.6 per 100,000 population over 55 years, and pertrochanteric fracture was the most commonly encountered hip fracture, accounting for up to 73.5% of cases^5^.

Traditional management of pertrochanteric fracture often includes surgical fixation, typically with a proximal femoral nail^2^. Pertrochanteric fixation in patients with osteoporotic bone requires a particular time for fracture healing. This long duration results in prolonged limitation of the patient’s functions and increases the risks of complications, such as non-union and mechanical implant failure like screw cut-through or cut-out, excessive migration of blade, peri-implant fracture and implant breakage ranging from 2.6 to 13%^6–8^. These can lead to poor quality of life^9^. Therefore, prompting faster bone union can reduce pain, encourage early ambulation, quality of life, and reduce complications.

Teriparatide, a recombinant human parathyroid hormone (PTH) (1–34 amino acid receptor-binding fragment), is considered a potent medication for osteoporosis therapy^10^. According to the literature, intermittent teriparatide administration provides marked anabolic effects that accelerate chondrocyte recruitment and differentiation, enhancing early enchondral ossification, stimulating the proliferation and differentiation of osteoprogenitors and chondroprogenitors, as well as preventing osteoblast apoptosis^11–13^. In addition, teriparatide enhances callus formation and improves the mechanical strength of bone at the fracture site^14^.

Previous studies reported that teriparatide enhanced pertrochanteric fracture healing and early clinical outcomes with heterogeneity of data^15–18^. On the other hand, other studies reported that teriparatide did not positively affect pain reduction, improve radiographic fracture healing, or decrease the rate of postoperative complications^17,19^. Therefore, the benefits of teriparatide on fracture healing remain uncertain.

The present study evaluated whether administering early and short-term daily teriparatide could fasten radiographic fracture healing and improve postoperative clinical outcomes and performance outcomes.

## Material and methods

This study was a single-center, prospective, randomized, double-blinded, placebo-controlled trial (RCT) approved by The Institutional Reviewed Board (IRB) of The Faculty of Medicine, Chulalongkorn University, Bangkok, Thailand (IRB) (No. 690/57). The study protocol was registered at ClinicalTrials.gov (https://clinicaltrials.gov/), and the identification number was NCT03133195; Clinical Trials Registry date 28/04/2017. All methods and experimental protocols were carried out according to relevant guidelines and regulations. Informed consent was obtained from all patients who participated in the study. The study was supported by the Thailand Science Research and Innovation Fund Chulalongkorn University, HEA663000022.

From April 2020 to June 2022, patients aged over 50 with pertrochanteric fractures (AO/OTA 31-A2 and 31-A3) and undergoing surgery using a proximal femoral nail anti-rotation (PFNA) were included in this study. Exclusion criteria were patients with baseline nonfunctional ambulator, ambulator-dependent for physical assistance levels 2 and 1, according to Mehrholz et al.^20^, known hypersensitivity to teriparatide or its analog, secondary osteoporosis, serum calcium > 10.5 mg/dL or 2.6 mmol/L, the serum PTH > 70 pg/mL, vitamin D (25-hydroxyvitamin D) < 12 ng/mL, alkaline phosphatase (ALP) > 120 UL, creatinine clearance (CrCL) < 30 mL/min, continuous treatment with digoxin, concurrent treatment with any medications for treating osteoporosis within 12 months. All surgeries were performed by two surgeons (CA and CT) using a single device (Proximal Femoral Nail Anti-rotation (PFNA), Synthes, Switzerland). Surgical steps include closed fracture reduction with fluoroscopic assistance to achieve anatomical position, stabilization of the fracture site, and instrumentation with a PFNA, according to the surgical manual. The study participants were followed for 24 weeks. The primary outcome was the time of radiographic fracture union after surgery. The secondary outcomes were changes in the Harris hip score (HHS), performance-based tests, and bone mass density (BMD) after teriparatide or placebo treatment after 24-week follow-up.

### Randomization

Patients were randomized into two groups following completing the surgery using a computer-generated sequence by a surgeon not involved in the surgery (CT). In the teriparatide group, the patients were assigned to receive a teriparatide (Mega PTH, Mega Lifesciences, Australia) dose of 20 μg daily at 48 h postoperatively (postoperative day 2). In the placebo group, the patient received normal saline designed in a container mimicking a teriparatide package. Calcium carbonate at 1000 mg daily and vitamin D2 (ergocalciferol) at 20,000 IU weekly were administered to all patients. The patient or the caregiver recorded the time and date of teriparatide or placebo administration until 24 weeks postoperatively. All patients were not allowed to take any anti-osteoporosis medications until the last follow-up.

### Postoperative ambulation

During the hospital stay, all patients, except those with unstable medical conditions, were encouraged to sit ambulation the next morning, transfer to a wheelchair on the 2nd day, and toe-touch weight-bearing ambulation from the 3rd day after surgery. The pre-injury HHS assessment was made on the unaffected hip before the patient underwent surgery. The postoperative HHS was evaluated on the 3rd day, the 2nd week, the 4th week, the 6th week, the 12th week, and the 24th week. Two performance-based measurements, including a five-time sit-to-stand test (5 × SST)^21^ and time up-and-go test (TUGT)^22^, were evaluated in the 2nd week, the 4th week, the 6th week, the 12th week, and the 24th week. Complications related to surgery and drug adverse events were evaluated and recorded during every patient visit.

### Radiographic fracture union and BMD evaluation

Radiographic fracture union was evaluated by four blind evaluators (AT, SN, ST, and NJ) using Radiographic Union Score for Hip (RUSH) on true anteroposterior and lateral cross-table radiographs during all follow-up visits^23^, which were conducted in the 2nd, 4th, 6th, 12th, and 24th weeks postoperatively. Criteria of radiographic bone union included the RUSH > 18, according to the study of Frank et al.^24^, with a minimum of three agreements among the four raters. The baseline BMD of the spine and the contralateral hip was performed during the patient’s hospital stay as the baseline, and the second BMD was again performed in the 24th week using the Hologic dual-energy X-ray absorptiometry QDR 4500 (Hologic Inc., Waltham, MA, USA).

### Statistical analysis

The intention-to-treat analysis was used in both outcomes. The sample size calculation was based on the study of Rana et al.^18^, aiming for an 80% study power, a 0.05 alpha error, and a two-week difference in the fracture healing time as the effect size. The minimum sample size was calculated to be 25. Statistical analysis was performed using SPSS version 29 (SPSS Inc., Chicago, IL, USA). Descriptive statistics for parametric data were shown as mean, standard deviation (SD), and 95% confidence interval (95% CI). The Chi-square or Fisher’s exact test was used to compare the radiological assessment of fracture healing times and categorical variables. A one-way ANOVA test was employed for clinical outcomes and continuous variables. The level of statistical significance was defined at *p* < 0.05. The graphs were visualized by GraphPad Prism software version 8 (GraphPad Prism Software Inc., La Zolla, LA, USA). The datasets used and/or analyzed for this study are available from the corresponding author at reasonable request.

## Results

**Fig. 1:**
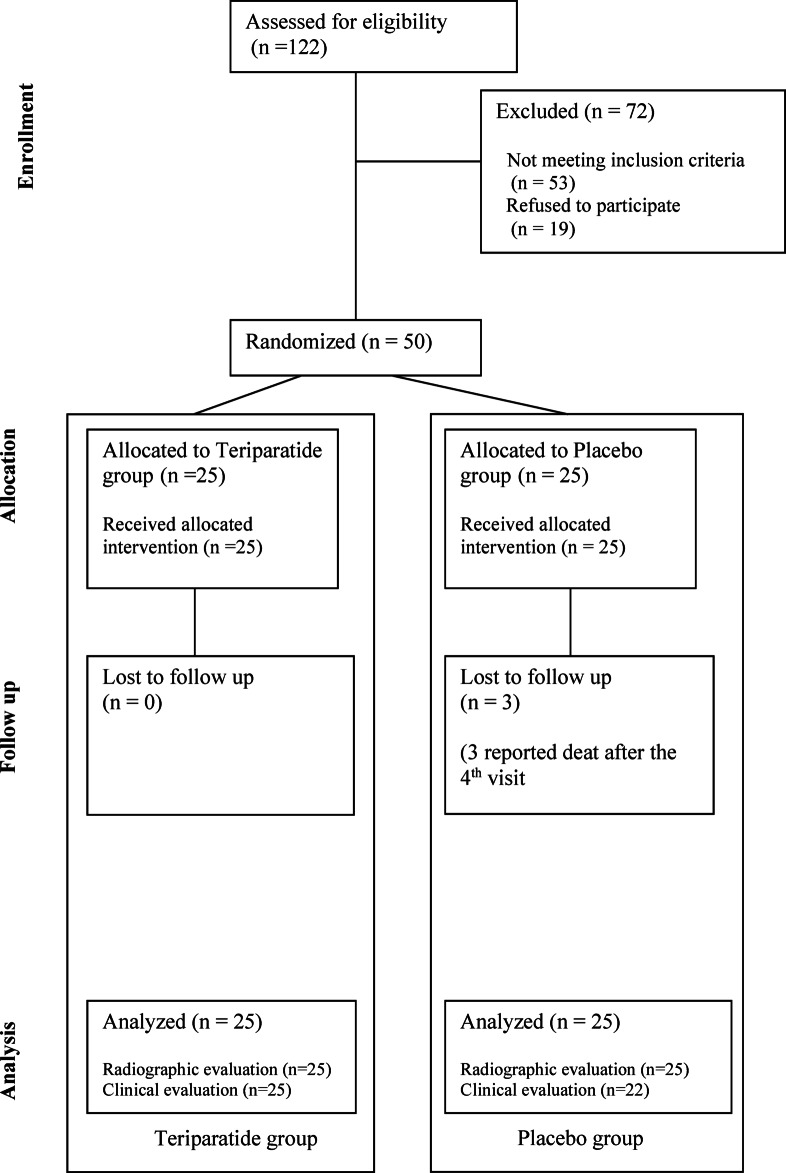
The CONSORT flowchart of the study.

A total of 122 patients were assessed for eligibility. Forty-six patients with serum PTH levels exceeding 70 pg/mL, ten patients with renal impairment, seven with current osteoporosis treatment, and nine patients denying participation in the study were excluded. Therefore, the remaining 50 patients were allocated for randomization. The Consolidated Standards of Reporting Trials (CONSORT) diagram is shown in Fig. 1. Patient demographics and baseline characteristics data are shown in Table 1. There were no statistically significant differences in baseline characteristics, including age, sex, AO/OTA fracture classification, affected side, HHS, BMD, and blood test results. During follow-up, three patients in the placebo group died after the 4th follow-up visit (the 12th week) due to underlying medical conditions not related to fracture treatment.

**Table 1:** Patient’s demographic data and baseline characteristics.

Parameters	Teriparatide group		Placebo group		*P* value
	N = 25		N = 25		
Age					
Mean ± SD (year)	72.9	± 7.5	72.6	± 7.9	0.97
Gender					
Female:Male (percent of female)	18:7 (72%)	18:7 (72%)	1		
Fracture AO/OTA classification					
31-A2	21		22		0.68
31-A3	4		3		
Side of affected hip					
Left:Right	10:15		07:18		0.55
Baseline clinical score					
HHS (mean ± SD)	78.21	± 15.51	75.4	± 16.68	0.59
Baseline BMD (mean ± SD)					
Spine (g/cm^3^)	0.798	± 0.157	0.785	± 0.345	0.79
Femoral neck (g/cm^3^)	0.518	± 0.156	0.517	± 0.201	0.80
Total hip (g/cm^3^)	0.631	± 0.211	0.598	± 0.220	0.37
Blood tests (mean ± SD)					
Calcium (mg/dL)	8.98	± 0.64	8.87	± 0.54	0.89
Albumin (g/dL)	3.69	± 0.56	3.55	± 0.45	0.43
Vitamin D level (ng/mL)	28.46	± 12.33	24.07	± 7.85	0.14
PTH level (pg/mL)	40.57	± 15.77	45.12	± 11.86	0.26
Creatinine (mg/dL)	0.95	± 0.40	0.90	± 0.35	0.50
Total protein (g/dL)	7.27	± 0.70	0.73	± 0.79	0.98

*AO/OTA* AO Foundation/Orthopaedic Trauma Association, *HHS* Harris Hip Score, *BMD* Bone mass density.

### Radiographic fracture union and BMD changes

**Fig. 2:**
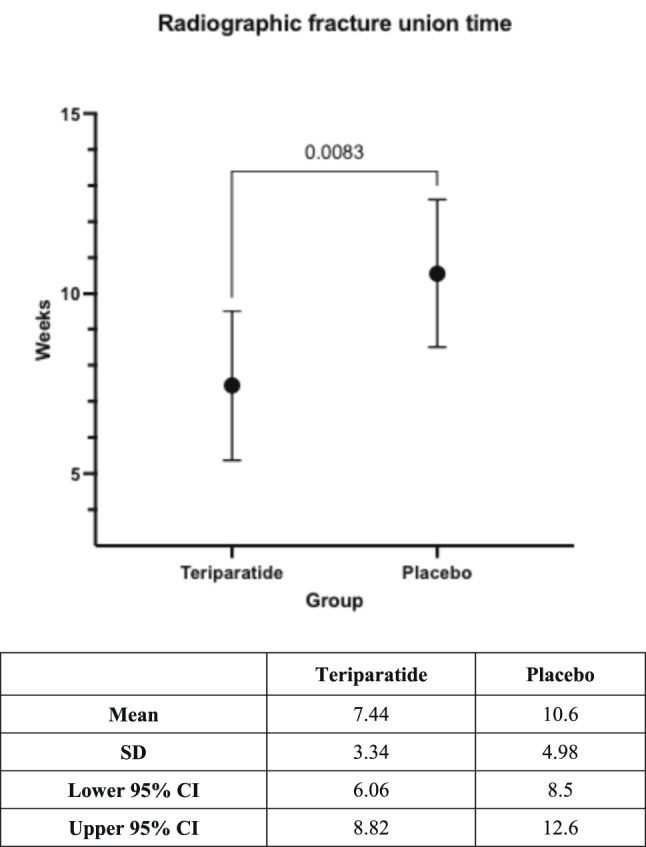
Graph and table comparing the means and 95% confidence intervals (95% CI) of the radiographic fracture union time between the teriparatide and the placebo groups demonstrated statistical significance.

The mean and SD of radiographic fracture union time of the teriparatide and the placebo groups were 7.44 ± 3.34 weeks (95% CI 6.06–8.82 weeks) and 10.56 ± 4.98 weeks (95% CI 8.50–12.62 weeks), respectively, with statistical significance (*p* = 0.0083) as shown in Fig. 2. The averaged RUSH at the time of fracture union of the teriparatide and the placebo groups were 24.4 points (range, 22.0–26.4 points) and 23.2 points (range, 22.0–25.6 points), respectively. None of the patients in both groups had a fixation failure, nor did the radiographs demonstrate bone non-union.

### Clinical and performance-based outcomes and changes of BMD

**Fig. 3:**
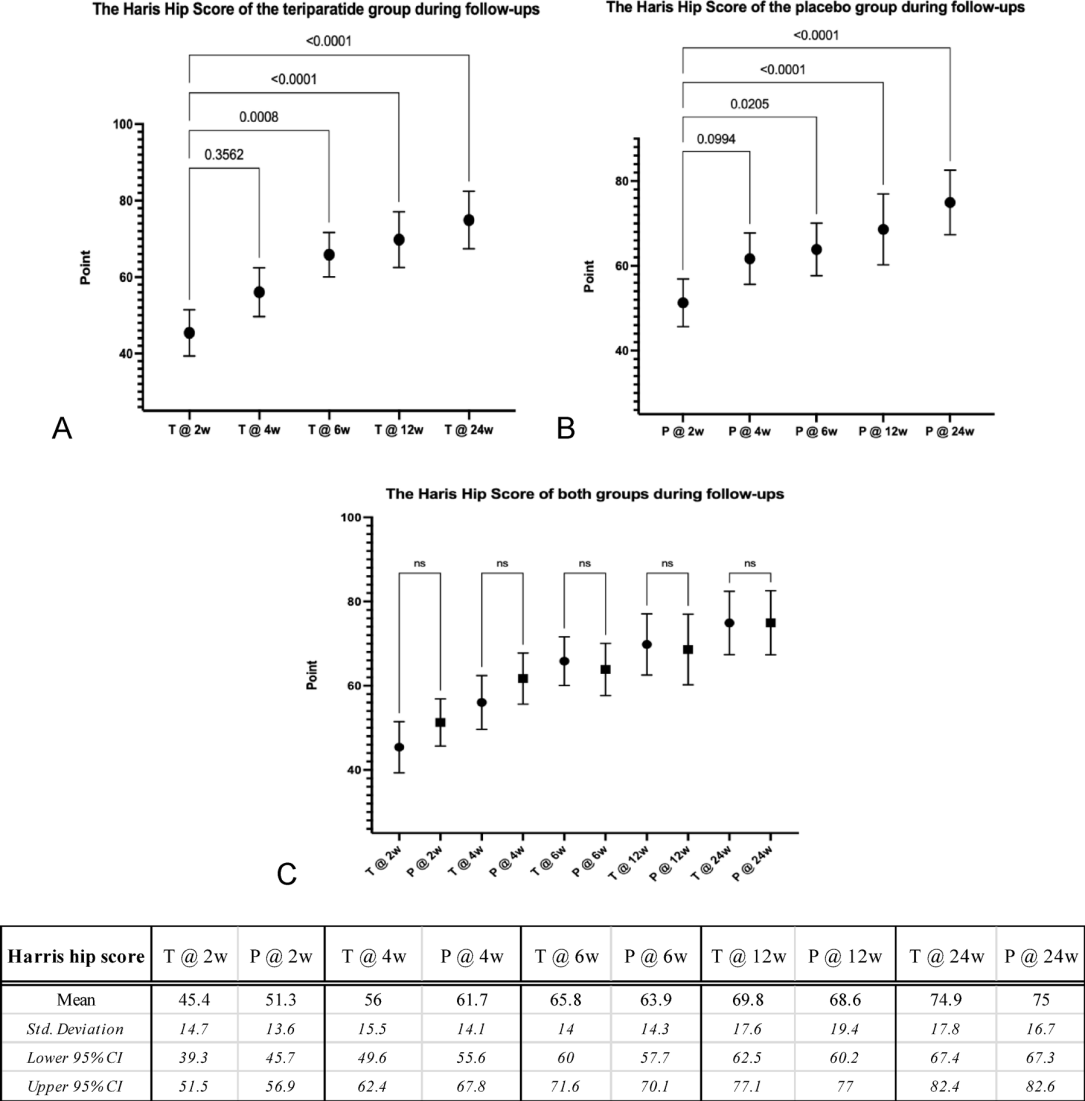
Graphs and table comparing means, standard deviations and 95% confidence intervals (95% CI) of the Harris Hip Score (HHS) between the teriparatide and the placebo groups during follow-ups evaluating with the 2nd week after surgery as the baseline. (**A**) The teriparatide group had significantly improved HHS from the 6th week and continued until the 24th week (*p* = 0.0008, < 0.0001 and < 0.0001, respectively). (**B**) The placebo group had similarly significantly improved HHS from the 6th week and continued until the 24th week (*p* = 0.0205, < 0.0001 and < 0.0001, respectively). (**C**) Improvement of HHS during follow-ups of both groups was not different; however, the significant level of the placebo group in the 6th week was lower *(p* = 0.0205 vs. 0.0008). (T: teriparatide group, P: placebo group).

The mean values of HHS of the teriparatide and the placebo groups at the 2nd, the 4th, the 6th, the 12th, and the 24th weeks post-surgery were 45.4, 56.0, 65.8, 69.8, and 74.9 points, and 51.3, 61.7, 63.9, 68.6 and 75.0 points, respectively. In the 6th week, both teriparatide and placebo groups had significantly improved HHS (*p* = 0.008 and 0.0205, respectively) compared to the 2nd week; the first-time patients could tolerate the clinical evaluation. The significant improvement of HHS continued until the 24th week (*p* < 0.0001 in both groups). Although there were no differences in improved HHS between both groups at all follow-up visits from the 6th week to the 24th week, the significant level of *p*-value in the teriparatide group at the 6th week was higher than in the placebo group (*p* = 0.0008 vs. 0.0205, respectively) as shown in Fig. 3.

**Fig. 4:**
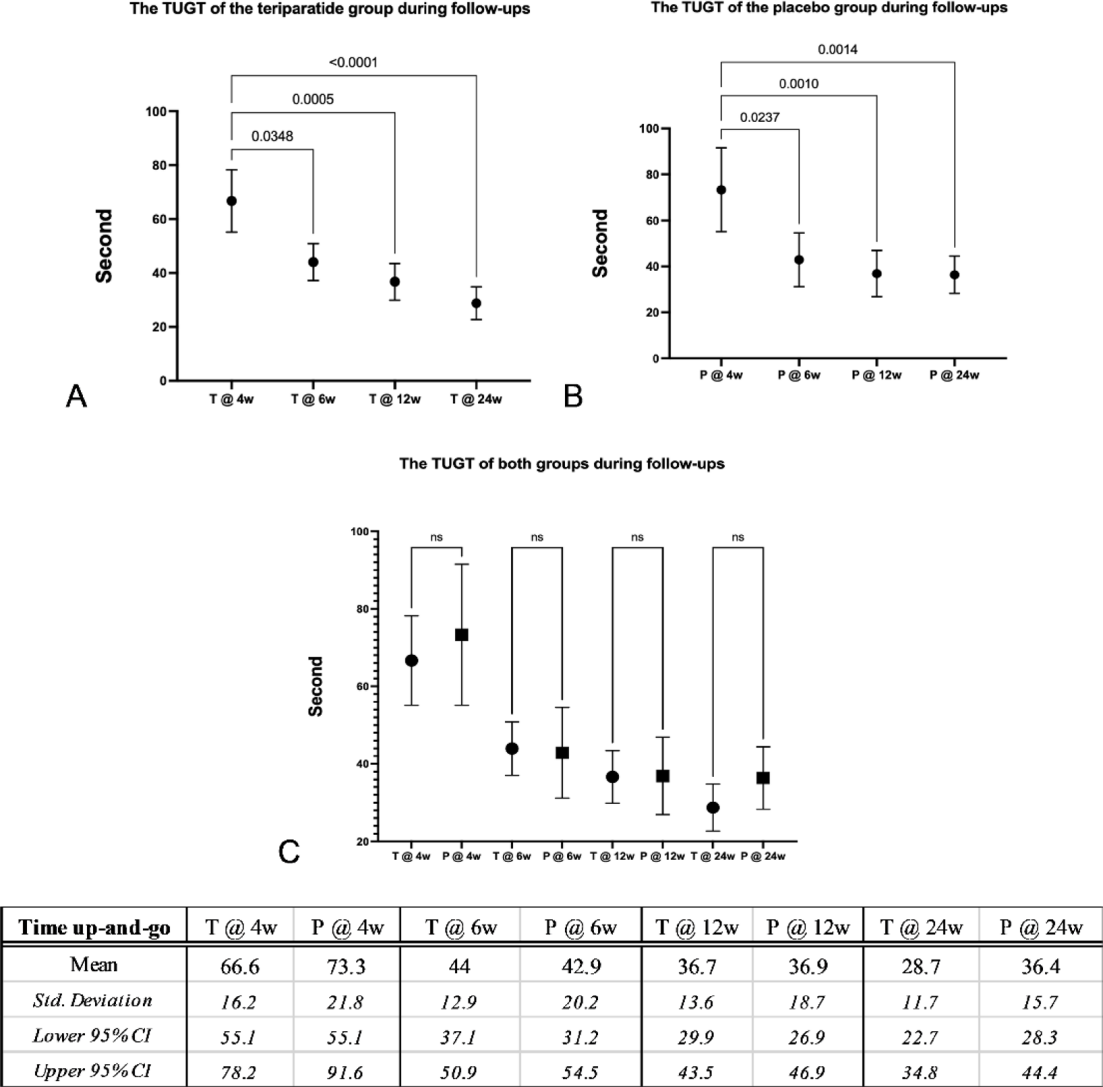
Graphs and table comparing means, standard deviations and 95% confidence intervals (95% CI) of the time up-and-go test (TUGT) between the teriparatide and the placebo groups during follow-ups with the 4th week after surgery as the baseline. (**A**) The teriparatide group had significantly improved TUGT from the 6th week and continued until the 24th week (*p* = 0.0348, 0.0005 and < 0.0001, respectively). (**B**) The placebo group had similarly significantly improved TUGT from the 6th week and continued until the 24th week (*p* = 0.0237, 0.001 and 0.0014, respectively). (**C**) Improvement of TUGT during follow-ups at all points of both groups was not different. (T: teriparatide group, P: placebo group).

The TUGT was able to be evaluated from the 4th week after surgery with the mean values at the 4th, the 6th, the 12th, and the 24th weeks of the teriparatide and the placebo groups of 66.6, 44.0, 36.7, and 28.7 s, and 73.3, 42.9, 36.9 and 36.4 s, respectively. Considering the 4th week as the baseline, both groups had significantly improved TUGT from the 6th week (*p* = 0.0348 vs. 0.0237, respectively) with no differences between both groups at all follow-up visits, as shown in Fig. 4.

**Fig. 5:**
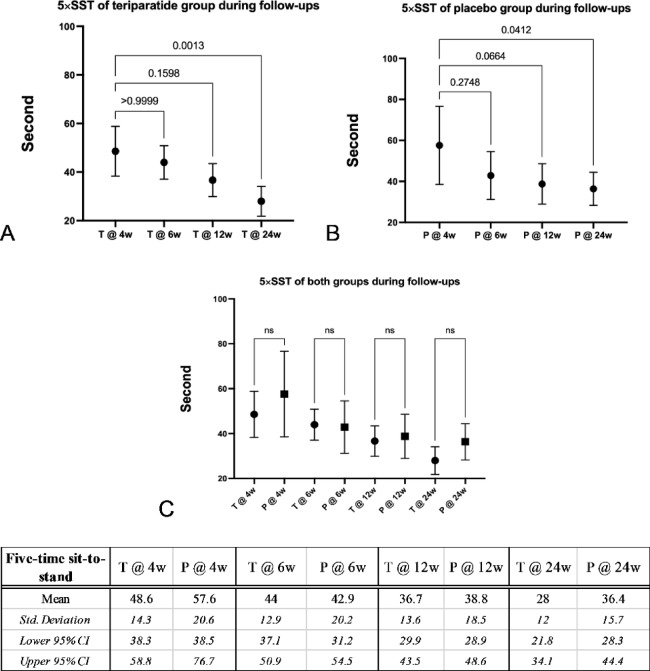
Graphs and table comparing means, standard deviations and 95% confidence intervals (95% CI) of the 5-time sit-to-stand test (5 × SST) between the teriparatide and the placebo groups during follow-ups evaluating with the 4th week after surgery as the baseline. (**A**) The teriparatide group significantly improved 5 × SST in the 24th week (*p* = 0.0013). (**B**) The placebo group similarly significantly improved 5 × SST in the 24th week (*p* = 0.0412), (**C**) Improvement of 5 × SST during follow-ups at all points of both groups was not different; however, the significant level of the placebo group in the 24th week was lower *(p* = 0.0412 vs 0.0013). (T: teriparatide group, P: placebo group).

Similar to the TUGT, the 5 × SST was able to be evaluated from the 4th week after surgery with the mean values at the 4th, the 6th, the 12th, and the 24th weeks of the teriparatide and the placebo groups of 48.6, 44.0, 36.7, and 28.0 s, and 57.6, 42.9, 38.8 and 36.4 s, respectively. Compared to the baseline in the 4th week, teriparatide and placebo groups significantly improved (*p* = 0.0013 and 0.0412, respectively). Although there were no differences at all follow-up visits, in the 24th week, the teriparatide group had better-improved 5 × SST than the placebo group, as shown in Fig. 5.

**Fig. 6:**
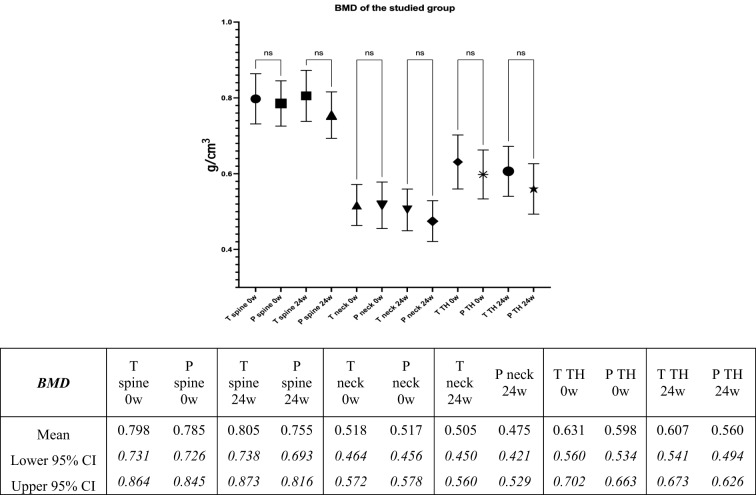
Graph and table comparing means and 95% confidence intervals (95% CI) of the changes in bone mass density (BMD) between the teriparatide and the placebo group during admission and in the 24th week demonstrated no statistical differences; however, the averaged bone loss of spine, femoral neck, and total hip of the placebo group were greater in the placebo group. (T: teriparatide group, P: placebo group, TH: total hip).

The mean BMD values of the total spine, femoral neck, and total hip of the teriparatide group and the placebo group at baseline were 0.798, 0.518, and 0.631 g/cm^3^ and 0.785, 0.517, and 0.598 g/cm^3^, respectively, with no statistical differences; however, at the 24th-week post-surgery, the averaged bone loss of the spine, femoral neck, and total hip was greater in the placebo group with no statistical differences from the teriparatide group, as shown in Fig. 6.

None of the patients had serious drug-related adverse events; however, one patient in the teriparatide group had a bruise around the injection site at the 2nd-week visit, and one patient in the placebo group had skin itching around the injection area. The mean serum calcium level at three months was 9.35 ± 0.68 mg/dL in the teriparatide group and 8.94 ± 0.68 mg/dL in the placebo group, with no statistically significant difference observed between both groups (*p* = 0.75).

## Discussion

The present study found that a short-term (12-week course), early administration of teriparatide could significantly fasten fracture union in pertrochanteric fracture fixation with an average of 3.12 weeks compared to placebo, while the HHS and TUGT significantly improved in both groups from the 6th week until the 24th-week post-surgery and the 5 × SST significantly improved in the 24th week. Although at the last follow-up (the 24th week), the improved TUGT and 5 × SST and the smaller decrease of BMD in the teriparatide group were better than the placebo group, there were no significant differences.

Although several studies evaluated the effect of teriparatide on osteoporotic hip fracture union after surgical fixation, there were high variations of study which could affect the outcomes, including the type of study, the device used for fracture fixation, the criteria for evaluating fracture union, and the method and the duration of teriparatide administration. Regarding observational studies, one study reported no positive result of adding teriparatide treatment after hip fracture fixation compared to those who underwent surgery alone^19^. Another study demonstrated improved postoperative functional outcomes and better fracture healing after hip fracture fixation^25^. Concerning only recent literature that focused on teriparatide’s effect on hip fracture union, seven RCTs were published, as shown in Table 2^18,26–31^. Three studies that were published earlier reported no difference in bone union time between the teriparatide and the control groups^26,27,31^; however, the control group in two of these studies was risedronate, which showed no differences in time to achieve fracture union at 12 weeks^27,31^. In contrast, four published studies later demonstrated teriparatide’s positive effect on accelerating fracture union compared to placebo^18,28–30^, of which one study started teriparatide from postoperative day 5^28^, and another study used a weekly teriparatide administered protocol^29^. Considering only three studies focusing on pertrochanteric fracture union following teriparatide^18,28,30^, all had a small sample size. The present study also had a small sample size but still had a higher number than those three studies. With strict selection criteria in the present study, several patients were excluded, mainly serum PTH > 70 pg/mL. Our finding was similar to the study of Chesser et al.^26^, reporting 80% exclusion of patients during screening due to high levels of PTH becoming contraindicated for teriparatide treatment. This finding implied that several hip fracture patients had osteoporotic bone with vitamin D deficiency and developed secondary hyperparathyroidism from vitamin D deficiency.

**Table 2:** List of published RCT studies comparing the radiographic outcomes between using and not using teriparatide.

Authors	Year of publication	Type of Study	Type of fracture	Selection criteria	Device for fixation	Follow up period	Teriparatide group	Duration of teriparatide treatment	Control group	Number of intervention vs control groups	Radiographic outcome
Chesser et al.^23^	2016	RCT	Pertrochanteric AO/OTA types 31-A1, 31-A2, and 31-A3	Age ≥ 60 years	DHS or cephalomedullary nail	12 months	Daily subcutaneous 20 μg within 10 days + Calcium and vitamin D	6 weeks	Calcium and vitamin D	15 versus 14	No radiographic comparision between two groups
Aspenberg. et al.^28^	2016	RCT	Pertrochanteric AO/OTA types 31-A1 and 31-A2	Age ≥ 50 years T score ≤ -2 SD	DHS or cephalomedullary nail	26 weeks	Daily subucutaneous 20 μg + Calcium and vitamin D + oral placebo weekly	8 weeks	Daily subcutaneous Placebo + Calcium and vitamin D + oral risedronate weekly	60 versus 65	No difference in bone union time, loss of reduction and implant failure
Malouf-Sierra et al.^24^	2017	RCT	Pertrochanteric AO/OTA types 31-A1 and 31-A2	T score ≤ -2 SD	DHS or cephalomedullary nail	78 weeks	Daily subcutaneous20 μg + Calcium and vitamin D + oral placebo weekly	78 weeks	Daily subcutaneous Placebo + Calcium and vitamin D + oral risedronate weekly	57 versus 61	No difference in union time, loss of reduction and implant failure
Rana. et al.^15^	2021	RCT	Pertrochanteric AO/OTA types 31-A1, 31-A2, and 31-A3	Age ≥ 50 years T score ≤ -2.5 SD	PFNA	6 months	Daily subcutaneous 20 μg + Calcium and vitamin D	24 weeks	Calcium and vitamin D	15 versus 15	Teriparatide had significantly shorter time to union
Mishra et al.^25^	2022	RCT	Pertrochanteric AO/OTA types 31-A1, 31-A2, and 31-A3	Age ≥ 50 years T score ≤ -2.5 SD	PFNA	6 months	Daily subcutaneous 20 μg from day 5 + Calcium and vitamin D	24 weeks	Calcium and vitamin D	16 versus 15	Teriparatide had significantly better RUSH score at 12 wk
Lee et al.^26^	2023	RCT	Femoral neck + pertrochanteric	Age ≥ 65 years	Cephalomedullary nail	12 months	Weekly subcutaneous 56.5 μg + Calcium and vitamin D	At least 12 weeks	Calcium and vitamin D	51 versus 41	Teriparatide had significantly better RUSH score at 3 and 6 months
Singh et al.^27^	2023	RCT	Pertrochanteric	Age ≥ 50 years	PFNA	6 months	Daily subcutaneous 20 μg + Calcium and vitamin D	24 weeks	Calcium and vitamin D	20 versus 20	Teriparatide had significantly shorter time to union
The present study	2024	RCT	Pertrochanteric AO/OTA types 31-A2 and 31-A3	Age ≥ 50 years	PFNA	6 months	Daily subcutaneous 20 μg within 2 days + Calcium and vitamin D	12 weeks	Daily subcutaneous Placebo + Calcium and vitamin D	25 versus 25	Teriparatide had significantly shorter time to union

*RCT* andomized controlled trial, *AO/OTA* Arbeitsgemeinschaft für Osteosynthesefragen/Orthopaedic Trauma Association, *DHS* dynamic hip screw, *PFNA* proximal femoral nail anti-rotation, RUSH Radiographic Union Score for Hip.

Due to the heterogeneity of several studies, the recent systematic review and meta-analysis did not support the idea that teriparatide enhances fracture healing after hip fracture fixation^17^. However, the present RCT study’s results proved teriparatide’s positive effect on the acceleration of fracture union after fixation of osteoporotic pertrochanteric fractures, which agreed with four previous RCTs^18,28–30^. As the present study had a short-term (12-week) course with early administration of teriparatide from postoperative day 2, which was defined as earlier administration and shorter duration than those previously reported without serious adverse events, it could be assumed that teriparatide for enhancing hip fracture union could be prescribed from postoperative day 2 and a 12-week course of treatment was proved effective.

Using the RUSH for evaluating fracture union was well accepted in the literature^16,23^, and both groups had no non-union after surgery. According to the surgical principle, all surgeries were performed with a carefully closed reduction to achieve an anatomical position with proper bone contact and fixation under fluoroscopic-assisted. Closed reduction to anatomical position and proper surgical fixation of the PFNA in osteoporotic pertrochanteric fracture played a key role in fracture union. In contrast, the teriparatide, administered postoperatively, played a supportive role in fastening the healing process, in which the average bone union time of the teriparatide group in the present study was 3.12 weeks faster than the placebo group.

Although abaloparatide, a new generation of parathyroid hormone agonists, is available in the market, teriparatide is still commonly used with physician-related familiarity and reasonable cost of treatment. The present study has some limitations. Firstly, the strict selection criteria for using teriparatide caused the exclusion of several patients with serum PTH > 70 pg/mL, which became contraindicated. Secondly, performance-based tests, including the TUGT and the 5 × SST, could not be evaluated before the fracture occurred, while the baseline of these measures could only be made when the patients could properly mobilize (from the 4th week onward); however, both tests reflected actual patients’ activities of daily living which were reliable for supporting radiographic bone union. Lastly, the duration of follow-up of this study was limited to 24 weeks; however, the time to the bone union as the primary outcome could be achieved in all cases, as well as the improvement of performance-based measures at the latest follow-up.

## Conclusion

A 12-week course with early administration of teriparatide post pertrochanteric fracture fixation fastened fracture union. Although there were no differences in the patient’s clinical outcomes at all follow-up time points, the teriparatide group had slightly better-improved HHS and TUGT in the 6th week and slightly better-improved 5 × SST with minimized declined BMD in the 24th week postoperatively.

## Electronic supplementary material

Below is the link to the electronic supplementary material.

Supplementary Material 1

## Data Availability

The datasets used and/or analysed during the current study available from the corresponding author on reasonable request.
